# Glomerular Endothelial Cell Crosstalk With Podocytes in Diabetic Kidney Disease

**DOI:** 10.3389/fmed.2021.659013

**Published:** 2021-03-24

**Authors:** Nassim Mahtal, Olivia Lenoir, Pierre-Louis Tharaux

**Affiliations:** Université de Paris, Paris Cardiovascular Center, Inserm, Paris, France

**Keywords:** podocyte, endothelium/physiopathology, diabetes, glomerulosclerosis, disease module identification, angiocrine factors, glycocalyx (glycocalix)

## Abstract

Diabetes is the main cause of renal failure worldwide. Complications of the kidney micro-and macro-circulation are common in diabetic patients, leading to proteinuria and can progress to end-stage renal disease. Across the complex interplays aggravating diabetes kidney disease progression, lesions of the glomerular filtration barrier appear crucial. Among its components, glomerular endothelial cells are known to be central safeguards of plasma filtration. An array of evidence has recently pinpointed its intricate relations with podocytes, highly specialized pericytes surrounding glomerular capillaries. During diabetic nephropathy, endothelial cells and podocytes are stressed and damaged. Besides, each can communicate with the other, directly affecting the progression of glomerular injury. Here, we review recent studies showing how *in vitro* and *in vivo* studies help to understand pathological endothelial cells-podocytes crosstalk in diabetic kidney disease.

## Introduction

Diabetes is a multifactorial disease and encompasses multi-organ complications, including kidney lesions leading to diabetic kidney disease (DKD). DKD is characterized by elevated urinary albumin excretion rate (UAER), increase in blood pressure, and decline in renal function leading to end-stage renal disease (ESRD). In addition, these patients have a high risk of cardiovascular disease, which further increases with deteriorating renal function. Although the methodologies to assess diabetes complications and consequences lack accuracy, possibly underestimating its burden, diabetes is recognized as major public health and economic plague (1). In parallel, the prevalence of diabetes in ESRD has been increasing constantly and diabetes is now the main cause of ESRD worldwide (2) and a rapidly increasing problem in the developing countries with the epidemic of type 2 diabetes. Albuminuria is an indicator of glomerular injury during diabetes, and a first step through ESRD (3).

The glomerular filtration barrier (GFB) is altered in DKD, a consequence of the combination of long-term hyperglycemia, advanced glycation end products through glycation reaction between reducing sugars, such as glucose, and proteins, lipids or nucleic acids; dysregulated insulinemia (with alternated hypo- and hyperinsulinemia), and frequently associated endothelial dysfunction and hypertension. Alterations of the GFB involve glomerular endothelial cells (ECs) and podocyte lesions. Endothelial dysfunction, increased extracellular matrix deposition, loss of podocyte permselectivity and progressive podocyte apoptosis occur along the time-course of DKD and contribute to the GFB dysfunction and progressive demise. Podocytes and ECs are physically close and isolated from each other by the glomerular basal membrane (GBM). ECs form a fenestrated endothelium delimiting the vascular compartment, whereas on the other side of the GBM podocytes board the urinary pole in the glomerulus. Communications between ECs and podocytes are physiological and occur from development to adult. During DKD however, pathological mechanisms such as hyperglycemia and hypertension impact the GFB, and lesions of the glomerular endothelium and the podocyte monolayer are common in DKD patients (4). EC are central players of the GFB, and damages of ECs participate to glomerulosclerosis and albuminuria in various pathological contexts, including diabetes (5–7). Evidence of a negative loop taking place between podocytes and ECs has been reported and reviewed, where stressed ECs impair podocytes and *vice versa* (8, 9). In this review, we focus on recent *in vitro* and *in vivo* data illustrating ECs-podocytes crosstalk in diabetic conditions.

### Endothelin-1 (ET-1) Is Detrimental for Podocytes and ECs in DKD

**Figure 1:**
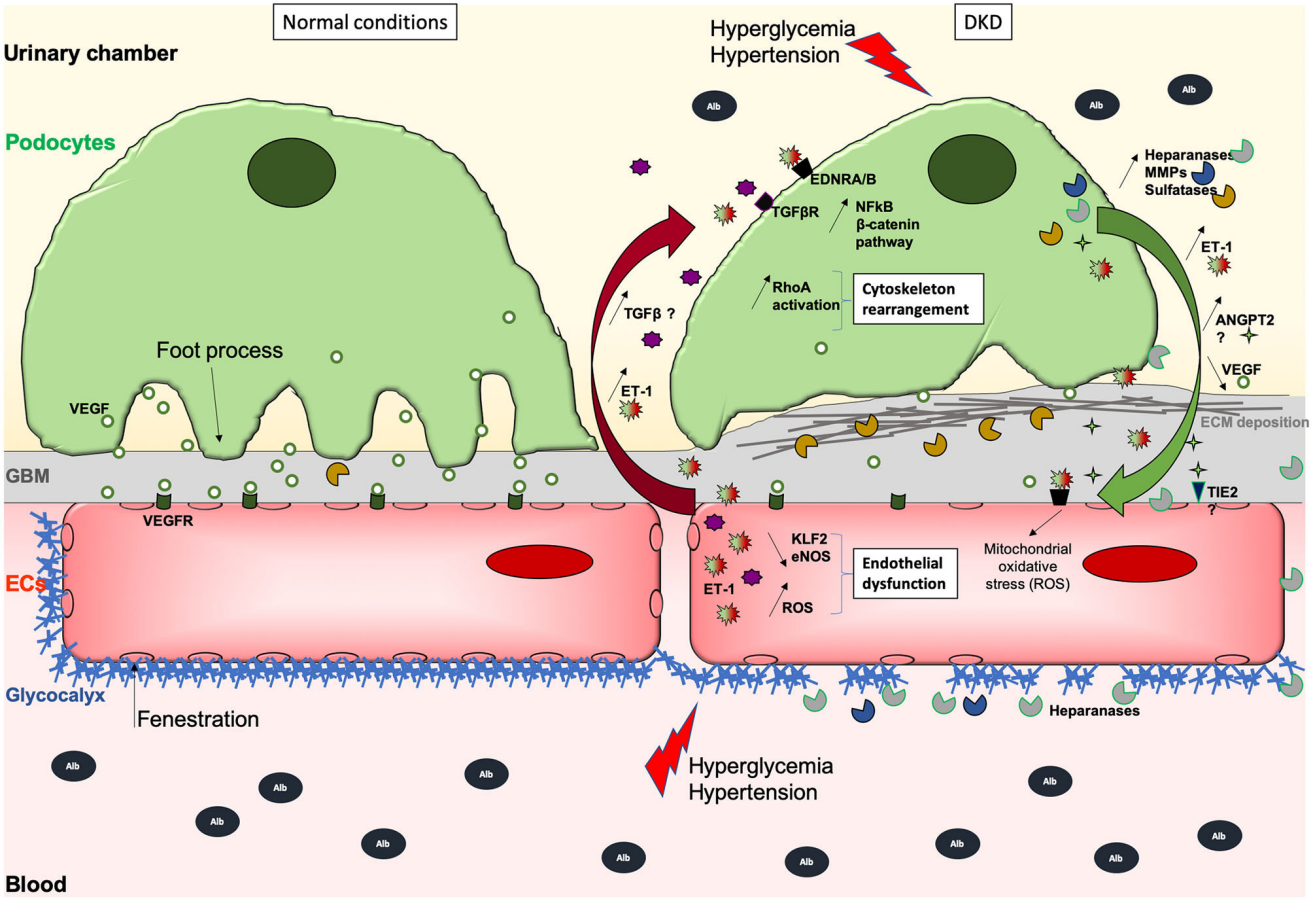
Schematic illustration of the GFB during normal (left) and DKD (right) conditions. During DKD, podocytes lose their foot processes, dedifferentiate with transiently increased ANGPT2 and VEGF production, and then detach or die, which then leads to fewer VEGF secretion. Initial and sustained loss of endothelial permselectivity is also fostered by ANGPT2/ANGPT1 imbalance fostering EC lesions through binding to its receptor TIE2. This initially reduces ECs quiescence and impairs permselectivity then ECs viability, and stimulates ET-1 synthesis that in turn acts on the other side of the GFB, activating podocyte Wnt/beta-catenin and NFkB pathways, heparanase release, inappropriate cytoskeletal remodeling, and abnormal extracellular matrix synthesis causing GBM thickening. ET-1 produced by EC but also by podocytes may also contribute to increased oxidative stress and secretion of proteases, which can degrade the glycocalyx, creating a vicious loop. Hence, ECs are subjected to oxidative and mitochondrial stress, lose their fenestrations, do not exert their functions properly, and may die. Deleterious effects of (ECs-derived?) -TGFβ-mediated signaling on podocytes are suggested. Moreover, the overproduction of sulfatases in the GBM could decrease the bioavailability of growth factors needed by ECs. The GBM thickens due to extracellular matrix (ECM) protein deposits, such as collagen fibers and fibronectin which augments the distance between rarefied podocytes and ECs, potentially altering podocyte to EC bidirectional signal cross-talk.

Endothelin-1 (ET-1) is a powerful vasoconstrictor and mitogen that has emerged as an interesting novel target for the treatment of DKD (10). ET-1 (EDN1) expression is increased in diabetic kidneys and higher plasmatic ET-1 levels are found in patients with diabetes as well as in animal models of DKD (11–14). ET-1 receptor blockers have renoprotective properties in several DKD (10, 15–17). ET-1 has a key role in regulating renal hemodynamics, salt and water homeostasis, and acid-base balance and in modulating cell proliferation, extracellular matrix accumulation, inflammation, and fibrosis. Consequently, any abnormality in the intrarenal ET system may result in renal dysfunction (e.g., salt sensitivity) and/or injury. Notably, the ET system is present in the renal areas targeted by diabetes, including the microvasculature, mesangial cells, and podocytes. To decipher whether the ET-1 system is a disease modifier beyond its role in the glomerular hemodynamics and sclerosis processes, our group investigated the roles of the ET receptors in podocytes in mice wherein podocyte-specific, double deletion of the ETA (EDNRA), and ETB (EDNRB) receptors was induced. These mice were protected against diabetes-induced podocyte loss and glomerulosclerosis but also provide evidence that the ETB receptor may play as important a role as does the ETA receptor. ETB receptor activation increased intracellular calcium and triggered the NF-κB and β-catenin signaling pathways, analogous to activation of the ETA receptor (Figure 1). The quantitative contribution of the ETB receptor may be substantial, as suggested by the fact that it is upregulated to a larger extent than the ETA receptors in the podocytes of diabetic mice. This study suggests an important role for it in mediating podocyte injury upon stimulation by ET-1, presumably produced by ECs during diabetes (18).

Recent publications from the Ilse Daehn group have also highlighted the role of ET-1 signaling in EC-podocyte crosstalk but with different mechanisms. They showed that when the forced expression of ET-1 by podocytes (and likely, in ECs) was induced through podocyte-specific activation of TGF-β signaling in transgenic mice and BALB/c mice with adriamycin-induced glomerulosclerosis, activation of ET-1 receptor type A in ECs induced mitochondrial oxidative stress and dysfunction, which in turn lead to release of yet unidentified factors mediating injury and depletion of podocytes in such experimental focal and segmental glomerulosclerosis (19). Glomerular endothelial mitochondrial dysfunction was also associated with increased glomerular ET-1 receptor type A expression and increased circulating ET-1 in experimental DKD. Moreover, pharmacological prevention of EC mitochondrial stress in this diabetes model prevented podocyte loss (20). Secreted factors from dysfunctional ECs were sufficient to cause podocyte apoptosis in supernatant transfer experiments or co-culture but this did not occur when ECs had been previously treated with mitoTEMPO, a mitochondrial antioxidant (21). Thus, ET-1 seems to be a key mediator in podocytes-to-ECs and ECs-to-podocytes communications promoting cell injury in several renal pathologies including DKD. In line with these experimental studies, the SONAR trial suggested that the ETA receptor antagonist atrasentan decreases albuminuria and the risk of major kidney outcomes when given to adults with type 2 diabetes, estimated glomerular filtration rate (eGFR) 25–75 mL/min per 1.73 m^2^, and a urine albumin-to-creatinine ratio (UACR) of 300–5,000 mg/g who had received maximum labeled or tolerated renin–angiotensin system inhibition for at least 4 weeks (10). Interestingly, albuminuria decrease with atrasentan was consistent irrespective of sodium glucose cotransporter 2 inhibitor (SGLT2i) use before enrolment in the SONAR trial, suggesting that the effects of atrasentan are additive to SGLT2i (22).

### Glomerular Glycocalyx Degradation in DKD

In glomeruli, the glycocalyx surrounding endothelial cells creates a space between the blood and the endothelium, controlling vessel permeability, restricting leukocyte and platelet adhesion, and allowing an appropriate endothelial response through mechanosensing. The negative charge of the glycocalyx on podocytes also repulses proteins, contributing to permselectivity towards negatively charged plasma proteins that limit their leakage in the urine. A study of Pima Indians with type 2 diabetes found that both podocyte damage and glomerular endothelial injury were commonly present in a cohort with macroalbuminuria. Interestingly, compared with podocyte injury, endothelial abnormalities were more closely associated with increased urine albumin excretion, suggesting that endothelial cell injury may be more critical to glomerular alterations in DKD compared with the commonly viewed importance of podocyte injury. Glycocalyx composition includes proteoglycans, glycoproteins, glycolipids, and glycosaminoglycans. Increased expression of proteolytic enzymes such as MMP9 (23–25), hyaluronidase (26), or heparanase [reviewed in van der Vlag and Buijsers (27)] was observed in diabetic patients and could participate in glycocalyx degradation in such pathological context, thus promoting proteinuria in diabetic patients (28, 29). MMP9 is mainly produced by podocytes and parietal epithelial cells in DKD where it participates in podocyte injury and promotes extracellular matrix (ECM) synthesis (23). Interestingly, MMP9 also promotes syndecan-4 shedding at ECs cell surface (30) and MMPs inhibition in a mouse diabetic model prevents syndecan-4 degradation and glycocalyx disruption in glomeruli (31). Whether it is the production by podocytes or by other cell types that induce ECs glycocalyx degradation in this context remains to be explored.

Heparanase is strongly up-regulated in podocytes exposed to high glucose (32, 33) (Figure 1). The increased heparanase expression by podocytes in kidneys has been demonstrated in DN (32, 34), and is essential for the development of albuminuria DN in both animal models and likely, in human (35, 36). Mice that lack heparanase develop less proteinuria or structural injury in diabetes induced with streptozotocin. Notably, loss of glycocalyx has been suggested in patients with type I diabetes 1 (37). Further, the development of microalbuminuria in diabetic patients results in further reductions of the systemic glycocalyx, leading to systemic vascular dysfunction (38). In Pima Indians with type 2 diabetes, podocyte foot processes in microalbuminuric participants were not different from those in control subjects and although microalbuminuria in type 2 diabetic Pima Indians often heralds progressive glomerular injury, it is not the result of defects in the size permselectivity of the glomerular barrier but rather of changes in either glomerular charge selectivity or tubular handling of filtered proteins or of a combination of these two factors (39). Another interesting study confirmed more directly that glycocalyx is perturbed in individuals with type 2 diabetes mellitus, and oral glycocalyx precursor treatment improved glycocalyx properties (28).

Garsen et al. demonstrated that heparanase production by podocytes promotes heparan sulfate degradation and glycocalyx disruption at podocyte and the endothelial cell surface in diabetic context by using *in vivo* mouse models and *in vitro* podocyte-to-EC supernatant transfer approaches (40) (Figure 1). Interestingly, in this latter article, the authors demonstrated that diabetes-mediated heparanase production in podocytes is mediated by endothelin pathway activation in podocytes in response to ET-1 production by ECs. Eberfors et al. also found that ET-1 signaling mediates degradation of the glomerular endothelial glycocalyx in non-diabetic kidney disease *via* pathological crosstalk between activated podocytes and glomerular endothelial cells but with a different mechanism. Indeed, here the authors found increased heparanase and hyaluronoglucosaminidase gene expression in glomerular ECs in response to podocyte-released factors and to ET-1 (41). Boels et al. further confirmed the crucial role of the endothelin pathway on heparanase expression and glycocalyx injury. Atrasentan, an antagonist of the endothelin receptor ETA, prevented glycocalyx degradation in DKD through reduction of glomerular and endothelial heparanase expression, although the production of heparanase by podocytes was not specifically explored in this context (42).

### VEGF Family Pathway Dysregulation in Glomeruli During DKD

Podocytes act as pericyte-like cells to support ECs differentiation and notably produce VEGF which is crucial to maintain glomerular ECs differentiation. In a pioneer work published in 2008, Hirschberg et al. showed that VEGF is upregulated in podocytes after high glucose (HG) treatment (43). VEGF is sufficient to promote proliferation and tube formation of blood outgrowth EC (BOEC) through Flk-1 *in vitro*, and co-culture of podocytes and BOEC also enhances proliferation of the latter, hence emphasizing the role of podocyte-derived VEGF. These data suggested a podocyte role on angiogenesis during the early onset of diabetic nephropathy *in vivo* (44, 45). The role of the VEGF family *in vivo* has already been extensively reviewed elsewhere (46–51), and the growing evidence point to the role of VEGFA and VEGFC during DKD. VEGFA is mainly expressed by podocytes, can be alternatively spliced in different isoforms such as VEGFA165b, and bind the endothelial receptors VEGFR1 and VEGFR2 (Figure 1). Besides, other members of the family include VEGFB, VEGFC, VEGFD, and PlGF. VEGFC can act both on lymphatic and blood vessels through VEGFR3 and VEGFR2, respectively. *In vitro*, VEGFC protects glomerular ECs from the negative influence of VEGFA reducing their permeability, and podocyte VEGFC overexpression protects ECs during diabetes *in vivo* (52). Besides, VEGFA and diabetic conditions (in db/db mice) increase glomerular albumin permeability *ex vivo*, which is rescued by VEGFC treatment. VEGFA is increased in HG-cultured podocytes, and a direct axis TGFβ1/VEGFA/AP-1, terminating in Bcl2 reduction and podocyte apoptosis, has been described (53). In the same study, inhibition of VEGFA or AP-1 was beneficial for diabetic rats. However, VEGF-A165b improved the permeability of isolated diabetic human glomeruli, and diabetic mice treated with VEGF-A165b or having a specific podocyte-overexpression of it develop a less severe phenotype (54). Dysregulation of the VEGF pathway during DKD would be probably more complicated than just dysregulation of VEGF synthesis by podocytes (Figure 1). The Semaphorin 3-Neuropilin axis is an important regulator of podocyte-to-endothelial cells during development (55) and plasmatic and urinary Semaphorin 3 expressions are positively associated with DKD (56, 57). Furthermore, advanced glycation end-products suppress Neuropilin 1 expression in podocytes, thus promoting their migration *in vitro* (58). Podocyte-selective Semaphorin 3A overexpression exacerbates DKD in mice by remodeling podocyte cytoskeleton and ECM synthesis (59). In addition to Semaphorin 3A signaling, Neuropilin 1 plays an important role in endothelial cells *via* its binding to VEGFA. Nevertheless, Neuropilin 1 expression in glomeruli seems restrained to podocyte and decreased during diabetes in mice (58) and to our knowledge, no one has modulated Neuropilin 1 expression specifically in endothelial cells during DKD to explore its function in such pathological context. Neuropilin 1 receptor could function as an extracellular scaffold protein generating podocyte-endothelial cell cross-signaling during DKD. Together, these data illustrate the intertwined and multiple effects of the VEGF family during diabetes.

### Other Influences of Glomerular ECs on Podocyte Injury During DKD

Physiologically, loops of glomerular capillaries are subjected to mean laminar shear stress (LSS) estimated at 10–20 dyne/cm^2^ (60). Slater et al. have shown that glomerular ECs submitted to LSS have increased ERK5 pathway activation leading to high KLF2 expression, which promotes ET-1, NO, and eNOS secretion (61). High KLF2 expression has also been observed in human glomeruli *ex vivo*. However, using conditioned media transfer from ECs to podocytes, and a co-culture strategy, they also show that LSS-exposed ECs secrete factors reducing the resistance of a podocyte monolayer. Of note, KLF2 is reduced in ECs exposed to high glucose but increased by insulin (62). In the same work, the authors showed that mice deficient for KLF2 in ECs have an aggravated phenotype during diabetes, and more pronounced podocyte lesions associated with higher glomerular mRNA levels of *Vegfa, Flk1*, and *angiopoietin 2*, and lower *Flt1, Tie2*, and *angiopoietin 1* levels. These data highlight crosstalk between ECs and podocytes relying on KLF2 endothelial expression level, which promotes in basal conditions an anti-inflammatory phenotype and appears required for ECs-podocytes homeostasis (Figure 1).

One of the first works identifying EC-to-podocyte crosstalk in DKD came from Isermann et al. Indeed, they demonstrated that activated protein C (APC), which is regulated by endothelial thrombomodulin, is downregulated in DKD. They used gain-of-function and loss-of-function complementary approaches in diabetic mice to show that APC inhibits hyperglycemia-induced endothelial and podocyte mitochondrial-dependent apoptosis (63).

Yuen et al. unraveled the role of eNOS in the EC-podocyte crosstalk (64). Mice deficient in eNOS develop podocytopathy, although eNOS is expressed in endothelial cells, but not in podocytes (65, 66). Authors have observed a marked cytoskeleton rearrangement of podocytes treated with the serum of diabetic eNOS-deficient mice, which suggests a modulation of the RhoA family that controls cytoskeleton dynamics. Moreover, increased activation of RhoA in podocytes treated with supernatants from glomerular ECs exposed to high glucose and/or angiotensin II isolated from diabetic eNOS-deficient mice was observed, despite the reduction of RhoA activity when ECs were from control mice. Nevertheless, the modulation of RhoA in podocytes has been reported detrimental (67).

Transforming growth factor-beta (TGF-β) is a well-described mediator of renal fibrosis in DKD with pleiotropic effects on glomerular cells. It promotes mesangial cell hypertrophy and extracellular matrix deposition and induces endothelial and podocyte dedifferentiation or death in mouse diabetic models [Reviewed in Chang et al. (68) and Ghayur and Margetts (69)]. TGF-β1 and TGF-β2 may originate from several cell types in DKD (in particular mesangial cells and EC) and as a secreted molecule, it would not be surprising that TGF-β could promote glomerulosclerosis and GFB dysfunction in DKD through paracrine mechanisms and participate in cross-communication within the GFB. The mechanisms for such deleterious effects of TGF-β on the GFB cellular components, podocytes and EC, are still unclear, whereas this growth factor displays contrasting hypertrophic and survival actions on other cell types such as fibroblast, mesangial cells or vascular smooth muscle cells.

A recent publication from regret Detlef Schlondorff's group highlighted the role of BAMBI a negative modulator of TGF-β1 in DKD with specific roles in ECs and podocytes (70). Interestingly the authors demonstrated that in diabetes, selective EC-*Bambi* deletion induced podocyte injury similarly to a selective podocyte-*Bambi* deletion. Diabetes-induced podocyte loss was even more pronounced in the EC-*Bambi* KO than in the podocyte-*Bambi* KO mice. Similarly, endothelial-selective autophagy inhibition also promotes podocyte injury in DN, supporting the concept that ECs injury in DKD may be a crucial mediator of podocyte injury and underscoring the importance of glomerular crosstalk in DKD (71).

ECs communicate with podocytes through secreted proteins, but also *via* exosomes during diabetes. To our knowledge, only one work from Wu et al. demonstrates that during diabetes, ECs could negatively affect podocytes by releasing exosomes (72). In HG conditions, ECs undergo endothelial-to-mesenchymal transition, secrete more exosomes, and the latter are internalized by podocytes which increased the TGF-β1/Wnt/β-catenin pathway. Consistently, podocytes treated with HG-cultured EC-derived exosomes are more permeable to albumin *in vitro*. Activation of the Wnt/β-catenin pathway in podocytes during diabetes and other proteinuric kidney diseases is known to be detrimental (73–75), and leads to oxidative stress (76).

### Other Influences of Podocytes on Glomerular ECs

In recent work, Ngo et al. collected plasma samples to assess renal arteriovenous gradients (77). A positive correlation between testican-2 and eGFR, and an association between higher baseline testican-2 levels and slower decline of eGFR, were observed in cohorts of patients including diabetics. Interestingly, testican-2 is expressed by podocytes and glomerular basal membrane, and can increase glomerular EC tube formation and motility, but not proliferation, *in vitro*. Hence, secreted testican-2 from podocytes seems beneficial for glomerular ECs in a variety of chronic kidney diseases including diabetes. Of note, testican-2 is not expressed in mice (78), illustrating the need for *in vitro* models to study podocyte-EC crosstalks. More complex culture models have been investigated, being 3D tri-partite cell cultures or “glomerulus-on-chip” systems. Waters et al. used glomerular EC, mesangial cells, and podocyte to reproduce the glomerular filtration barrier in 3D cultures (79). They showed that TGFβ-induced glomerulosclerosis, as seen in DKD, was prevented in 3D tri-cultures by conjoint inhibition of ALK5 and CTGF, and differential effects of TGFβ on mesangial cells and glomerular ECs. TGFβ led to nodule formation and loss of ECs arborization in 3D tri-cultures, and in mono-cultures, it increased the mediator CTGF expression in podocytes and increased ALK5 expression in mesangial cells, which favors an upregulation of TGFβ pathway activation through SMAD2/3. Moreover, BMP7 appeared to modulate the effects of TGFβ on ECs but not in mesangial cells.

Another well-known angiogenic signaling is likely to be involved in podocyte-to-endothelial cell cross-communication in DKD, whereas direct evidence is missing. Angiopoietin 1 (ANGPT1) produced by podocytes promotes maturation and stabilization of glomerular capillaries *via* TIE2 receptor on endothelial cells (80), playing an important role in the regulation of angiogenesis, endothelial cell survival, proliferation, migration, adhesion, and spreading, but also maintenance of vascular quiescence. Angiopoietin 2 (ANGPT2) has opposite effects to ANGPT1 by destabilizing blood vessels and effects of ANGPT2 are dependent on VEGFA levels (81, 82). ANGPT2 levels are associated with indexes of endothelial dysfunction in clinical diabetes mellitus (83–85). A decreased circulating ANGPT1/ANGPT-2 ratio may contribute to the development of DKD after administration of STZ in mice (86) and STZ-induced DKD rats (87). Meanwhile, plasma ANGPT2, like VEGF, was found to be raised in human diabetes regardless of vascular disease. Whereas, both growth factors correlated with HbA1c and with each other, ANGPT2 levels did not correlate with carotid atherosclerosis, plasma von Willebrand factor (vWf), and urine albumin to creatinine ratio in humans with type 2 diabetes after multiple adjustment (88). This is not surprising as the ANGPT system is regulated locally in the microvessels. Regulation of glomerular angiopoietin levels is key in animal models of DKD. To further investigate the role of ANGPT1 in diabetes, Jeansson et al. compared diabetic controls and *Angpt1*-deleted mice induced with STZ. The *Angpt1* knockout kidney showed accelerated diabetes-mediated glomerular damage, suggesting that ANGPT1 could potentially protect the glomerular microvasculature from diabetes-induced injury (89). Mice with podocyte-specific inducible ANGPT-1 overexpression in the early stage of DKD led to a 70% reduction of albuminuria and prevented diabetes-induced GEC proliferation via increased TIE-2 phosphorylation suggesting a critical role of ANGPT1-1/ANGPT-2 in early DKD. Meanwhile, hyperfiltration and renal morphology were unchanged, indicating a still limited role (90). The role of angiopoietins in DKD has been nicely reviewed elsewhere (91, 92). Overall, the authors consider that angiopoietins are produced by podocyte and signal *via* TIE2 in endothelial cells only, which is most likely the mechanism but has not been entirely demonstrated.

Finally, dysregulation of the GBM composition by podocytes could also impair ECs in DKD. Indeed, heparan sulfate, the major component of glomerular ECM, modulates growth factor signaling, notably modulating VEGFA availability to the surrounding ECs. Schumacher et al. showed that Wilms' Tumor 1 changes VEGFA and FGF2 signaling by increasing the expression of the 6-*O*-endosulfatases Sulf1 and Sulf2, which remodel the heparan sulfate 6-*O*-sulfation pattern in the GBM (93). Mice deficient in both *Sulf1* and *Sulf2* developed age-dependent proteinuria as a result of ultrastructural abnormalities in podocytes and endothelial cells. Sulf1 and Sulf2 double-knockout (DKO) mice also showed glomerular hypercellularity, matrix accumulation, and GBM irregularity. Platelet-derived growth factor (PDGF)-B and PDGF receptor-β were upregulated in Sulf1 and Sulf2 DKO mice. Diabetic mice showed an upregulation of glomerular Sulf1 and Sulf2 expression and diabetic Sulf1 and Sulf2 DKO mice showed an acceleration of the glomerular pathology without glomerular hypertrophy (94). Thus, Sulf1 and Sulf2 may play protective roles in DKD, probably by modulating growth factor availability to podocytes and ECs.

## Discussion

Across the years, researchers tried to model pathological conditions happening during DKD, by culturing EC and/or podocytes with HG. More recently, this EC-podocyte crosstalk has been explored with conditioned media transfers. This shed light on the crucial role of VEGF, produced by podocytes for EC survival and dysregulated during DKD (43, 50, 95), but also to signaling pathways modulated in EC or podocytes during the excess of glucose. In parallel, rodent DKD models were developed, confirmed both of these observations, and even more. Transgenic animals gave substances to the former hypothesis and opened new horizons, as integrated systems (96). Nevertheless, cell cultures remain powerful tools, they tend to naturally evolve from monolayered and mono-typed to multi-typed, organoids and even glomerulus-on-chip, to represent an alternative, or at least an intermediate, to *in vivo* studies. Recently, Wang et al. developed a tri-partite glomerular filtration barrier in a 3D culture from rat glomeruli and showed that high glucose conditions in the endothelial side increased barrier permeability and podocyte migration in this model (97). Zhou et al. developed a device composed of ECs and podocytes cultured together but physically separated by a porous membrane (98). Hypertensive conditions on the endothelial compartment led to increased barrier permeability and damages of both EC and podocytes. Hence, *in vitro* models constitute an efficient way to study glomerular ECs – podocytes crosstalk during DKD. More importantly, they are useful to dissect molecular pathways involved in pathophysiology. Recent advances in 3D cell cultures and microfluidics tend to combine the comfort and high throughput of *in vitro* assays with partial biological relevance of *in vivo* studies. A significant limitation of such 3D systems being obviously to poorly mimic the pathophysiological environment of glomerular cells as would occur in chronic diabetic condition.

ECs - podocytes crosstalk is crucial during DKD development, where ECs also dialogue with other renal cell types. As an example, the elevation of EC-secreted ET-1 directly enhances mesangial expansion and extracellular matrix deposits, characteristics of DKD (99). ECs injury could also participate in parietal epithelial cell activation, a condition seen in focal segmental glomerulosclerosis (FSGS), a rare-to-common renal complication of diabetic patients. Indeed, Luque et al. demonstrated that Hif2α pathway inhibition in endothelial cells only sensitized mice to the development of hypertension-induced FSGS, suggesting that signals from the ECs could be transferred to PEC (100). Finally, renal tubular cell injury in diabetes modulates ECs [reviewed in Chen et al. (101)].

Together, these studies showed through different methodologies, from cell cultures to human samples, that glomerular ECs are crucial actors of DKD pathophysiology, and cross-communications with podocytes constitute major events for diabetic renal disease progression. Intensive control of glucose and blood pressure along with RAS inhibition and SGLT2i (for patients with type 2 DM) remain the clinical gold standards to deter the progression of DKD. The armament may be complemented in the near future with glucagon-like peptide 1 (GLP-1) receptor agonists, non-steroidal selective mineralocorticoid receptor antagonists (MRAs) or ETRAs. In fact, metformin, RAAS and ET-1 inhibitors were shown to prevent endothelial dysfunction beyond their effect on insulin resistance. Recent anti-diabetic drugs also display clear effects on the microcirculation in animal models and patients (102–107). Additional work is needed to understand the mechanisms involved, and new treatments that aim to prevent microvascular injury or restore microvascular function could be an effective strategy for preventing; or even reversing DKD. Such strategies may consider crosstalk within the glomerular system. Fine tuning of angiogenic systems and cellular energetics, promotion of autophagy, of glycocalyx protection, alleviation of chronic sterile inflammation and senescence may offer promising perspectives. The variety of molecules involved represents as many potential therapeutic targets to better take charge of the DKD burden and improve patient lives. Future studies need to consolidate the concept of the glomerulus as an integrated functional unit.

## Author Contributions

NM, OL, and P-LT wrote the manuscript. All authors contributed to the article and approved the submitted version.

## Conflict of Interest

The authors declare that the research was conducted in the absence of any commercial or financial relationships that could be construed as a potential conflict of interest.
